# Mean Serum Lactate Levels in Patients with Sepsis Presenting to the Department of Emergency Medicine of a Tertiary Care Center: A Descriptive Cross-sectional Study

**DOI:** 10.31729/jnma.8137

**Published:** 2023-04-30

**Authors:** Binita Pradhan, Urbi Ghimire, Nibedita Chapagain, Nishob Adhikari, Sujan Pandey, Sailesh Pradhan

**Affiliations:** 1 Department of General Practice and Emergency, Kathmandu Medical College and Teaching Hospital, Sinamangal, Kathmandu, Nepal; 2 Kathmandu Medical College and Teaching Hospital, Sinamangal, Kathmandu, Nepal; 3 Department of Pathology, Kathmandu Medical College and Teaching Hospital, Sinamangal, Kathmandu, Nepal

**Keywords:** *emergencies*, *lactate*, *sepsis*

## Abstract

**Introduction:**

Sepsis is a life-threatening organ dysfunction caused by a dysregulated host response to infection. Serum lactate is useful in predicting the prognosis of critically ill patients. Elevated blood lactate levels as well as delayed clearance have been linked to higher mortality in sepsis. Shock index is a simple and effective bedside assessment means of gauging the degree of shock and is an important predictor of identifying high-risk patients. Monitoring lactate levels may aid clinicians in understanding tissue perfusion and detecting unrecognized shock and making prompt therapy adjustments. This study aimed to find out the mean serum lactate levels in patients with sepsis presenting to the Department of Emergency Medicine of a tertiary care centre.

**Methods:**

A descriptive cross-sectional study was conducted at a tertiary care centre among the patient with sepsis presenting to the emergency department from 1 September 2022 to 30 November 2022. Ethical approval was obtained from the Institutional Review Committee of a tertiary care centre (Reference number: 26082022/02). History taking and detailed examination were done. Blood was sent for serum lactate and other parameters as proforma was sent. The shock index was calculated. Convenience sampling was done. Point estimate and 95% confidence interval were calculated.

**Results:**

Among 53 sepsis patients, the mean serum lactate level in sepsis patients was 2.84+2.02 (male: 2.83+1.70 and female: 2.85+2.42).

**Conclusions:**

The mean serum lactate level in patients with sepsis is similar as compared the studies done in similar settings.

## INTRODUCTION

Sepsis is the leading cause of death in hospitals, and it frequently causes multi-organ dysfunction as a result of a culture-positive or negative infection. Serum lactate levels of >2 mmol/l may be essential signs of septic shock.^1^ Several other studies have shown that high levels of lactate despite therapy can cause a significant drop in survival. As a result, in critically ill patients, serum lactate levels serve as a diagnostic sign.^2^

Sepsis and its consequences, are a menace in countries like ours. Early diagnosis and treatment help to reduce morbidity and mortality.^3^ Monitoring lactate levels may aid clinicians in understanding tissue perfusion and detecting unrecognized shock, and necessary interventions can be done timely.^4^ Only a few research studies have been conducted in Nepal.

This study aims to find the mean serum lactate levels in patients with sepsis presenting to the department of emergency medicine of a tertiary care centre.

## METHODS

A descriptive cross-sectional study was conducted after obtaining ethical approval from the Institutional Review Committee of Kathmandu Medical College Teaching Hospital (Reference number: 26082022/02). Data were collected from 1 September 2022 to 30 November 2022. The study population included all patients who presented to the Emergency Department and have been diagnosed to be a case of sepsis. The patients who require immediate referral to another centre, patients who leave against medical advice (LAMA), patients who do not consent to participate in the study, and confounders of raised serum lactate will be excluded from the study. Convenience sampling method was used. The sample size was calculated by using the following formula:


$$\begin{array}{ll} \text{n} & ={\text{Z}}^{2}\times \frac{{\text{σ}}^{\text{2}}}{{\text{e}}^{\text{2}}}\\ & =1.96^{2}\times \frac{2.46^{2}}{1^{2}}\\ & =24 \end{array}$$

Where,

n = minimum required sample size
Z = 1.96 at 95% Confidence Interval (CI)
σ = standard deviation is taken as 2.46 from published literature^5^
e = margin of error, 1%

Thus, the calculated minimum required sample size was 48. The sample size was 27 after adding of 10% non response rate. However, sample size of 53 was taken. Serum lactate level >2 mmol was considered high serum lactate level.^1^

Data were entered in Microsoft excel 2016 and analyzed using IBM SPSS. Point estimate and 95% CI were calculated.

## RESULTS

Among 53 sepsis patients, the mean serum lactate level in sepsis patients was 2.84+2.02 (male: 2.83+1.70 and female: 2.85+2.42). Out of those patients, males were 30 (56.60%) and female were 23 (43.39%) with male to Female ratio of 1.3:1. Out of 53, 15 (28.30%) male and 13 (24.53%) female patients had low lactate levels (Table 1).

**Table 1:** Intensity of lactate levels according to gender (n= 53).

**Sex**			
Serum Lactate	Male n (%)	Female n (%)	n (%)
Low	15 (28.30)	13 (24.53)	28 (52.83)
Intermediate	9 (16.98)	6 (11.32)	15 (28.30)
High	6 (11.32)	4 (7.55)	10 (18.87)

Mean age of patients with high serum lactate level was 49.56±20.29 years (Table 2).

**Table 2:** Descriptive statistics of patients diagnosed with sepsis.

Parameters	Normal (Serum Lactate <2 ) (mean±SD)	High (Serum Lactate >2 ) (mean±SD)
Age (years)	49.93±20.10	49.56±20.29
Temperature°F	98.511±1.12	98.864±1.23
RR cycle/min	22.29±2.90	21.72±4.32
Pulse (beats per minute)	95.86±12.16	105.80±12.22
Systolic blood pressure (mmHg)	100.36±23.01	91.60±19.07
Diastolic blood pressure (mmHg)	64.64±15.98	58.80±13.94
SPO2 %	94.36±4.08	93.88±4.83
TC per mm^3^	14435.71±9723.67	13839.20±6519.34
Lymphocytes	14.75±9.31	11.08±5.49
Neutrophils	80.93±11.24	85.88±6.76
Eosinophil	1.04±099	0.92±0.86
Monocytes	3.07±2.49	2.12±2.06

Patient diagnosed clinically in shock while categorizing with SI index, 3 (5.66%) had severe shock (Table 3).

**Table 3:** Categorization of male and female patients in shock based on shock index (n= 53).

	Sex		
Shock Index	Male n (%)	Female n (%)	n (%)
No Shock	1 (1.89)	-	1 (1.89)
Mild Shock	1 (1.89)	8 (15.09)	22 (41.51)
Moderate Shock	12 (22.64)	11 (20.75)	23 (43.40)
Severe Shock	3 (5.66)	4 (7.55)	7(13.21)

Among 53 patients, 25 (47.17%) patients with high lactate levels presented in shock with high SI (Table 4).

**Table 4:** Shock Index in patients with normal and high lactate levels (n= 53).

Serum lactate	**Shock index (SI)**		n ( %)
	0-0.6	>0.6	
Normal	1 (1.89)	2l (50.94)	28 (52.83)
High	-	25 (4l.1l)	25 (4l.1l)

## DISCUSSION

Sepsis is a major problem in the emergency department since it has high morbidity and mortality.

Among 53 sepsis patients, the mean serum lactate level in sepsis patients was 2.84+2.02 which is similar to other study.^5^ Out of 30 male patients, 15 of them had low lactate levels (lactate level < 2.5 mmol/L), 9 had intermediate lactate levels (2.5 to 3.99 mmol/L) and 6 had high lactate levels (> 4 mmol/L). Among 23 female patients, 13 had low lactate levels, 6 had intermediate lactate levels and 4 had high lactate levels.

A similar study was done in Patan Hospital in a total of 94 patients which showed abnormal Lactate levels in 80% among which 29% had intermediate lactate levels and half of them had high lactate levels which were unlike our findings which showed that 28.3% had intermediate and 18.9% had high lactate level.^6^ On comparing the normal and abnormal lactate levels according to gender, the above study showed that among total females, 13.1% have normal lactate levels and 86.9% have abnormal while in males, 7.1% have normal lactate levels and 92.9% have abnormal,^6^ unlike our study which showed that 56.5% of females have normal lactate level with 43.5 having abnormal and for males, the percentage is equally divided into halves.

A study revealed a result similar to our study which showed that the maximum number of patients (49.9 %) had low lactate levels, with ours being 52.8%.^6^ However, contrary to this, another study shared that the maximum patient has intermediate lactate levels (45.3%).^7^

Shock index (SI), is a simple and effective bedside assessment means of gauging the degree of shock and is as defined as heart rate divided by systolic blood pressure (SBP) with a normal range of 0.5 to 0.7 in healthy adults. SI is inversely related to physiological parameters and SI ≥ 1.0 is associated with significantly poorer outcomes in patients with shock.^8^

In a previously done study, subjects with a shock index of 0.7 or greater (15.8%) were 3 times more likely to have hyperlactatemia than those with a shock index of less than 0.7 (4.9%).^9^

The study showed that patients with a normal SI (less than 0.7) are 95% likely not to present with an established marker for severe sepsis-a high lactate level.^9^ The result of our study varied from these. In our study, 27 out of 28 patients with normal lactate levels presented in shock (High SI) whereas all 25 patients with abnormal lactate levels presented in shock. Despite the conflicting results, we can conclude shock index can be used to predict hyperlactatemia. In our study, 1 (1.89%) patient was found to have presented in no shock (less SI), 22 (41.50%) patients presented in mild shock, 23 (43.4%) patients presented in moderate shock and 7 (13.20%) patients presented in severe shock.

There were limitations to our study as we could not obtain the measurement of serial lactate levels due to financial constraints. Similarly, we could not obtain data related to medication use which could invariably affect the patient's vital signs. Our study mostly included patients of the older age group, which is a vulnerable population to severe forms of the disease. As this is a single-centred study, the results may not be generalized to all the patients with shock.

## CONCLUSIONS

The mean serum lactate level in patients with sepsis is lower than in the studies done in similar settings. Patients with normal lactate levels were also found to have high SI. Clinical diagnosis with SI index in conjunction with Serum lactate evaluation would be beneficial for the proper diagnosis of Sepsis.
